# Progress on the Elucidation of the Antinociceptive Effect of Ginseng and Ginsenosides in Chronic Pain

**DOI:** 10.3389/fphar.2022.821940

**Published:** 2022-02-21

**Authors:** Mei-Xian Li, Qian-Qi Wei, Huan-Jun Lu

**Affiliations:** ^1^ National and Local Joint Engineering Research Center of Technical Fiber Composites for Safety and Protection, Nantong University, Nantong, China; ^2^ Department of Infectious Diseases, General Hospital of Tibet Military Command, Xizang, China; ^3^ Institute of Pain Medicine and Special Environmental Medicine, Nantong University, Nantong, China

**Keywords:** chronic pain, ginseng, ginsenosides, antinociception, anti-inflammation

## Abstract

Ginseng (Panax ginseng C.A. Meyer) is a traditional Oriental herbal drug widely used in East Asia. Its main active ingredients are ginsenosides whose constituents are known to have various pharmacological activities such as anticancer, antinociception, and neuroprotection. The analgesic effects of ginsenosides, such as Rg1, Rg2, and Rb1, as well as compound K, are well known and the analgesic mechanism of action in inflammatory pain models is thought to be the down regulation of pro-inflammatory cytokine expression (TNF-α IL-1β, and IL-6). Several studies have also demonstrated that ginsenosides regulate neuropathic pain through the modulation of estrogen receptors. Recently, an increasing number of pathways have emerged in relation to the antinociceptive effect of ginseng and ginsenosides. Therefore, this review presents our current understanding of the effectiveness of ginseng in chronic pain and how its active constituents regulate nociceptive responses and their mechanisms of action.

## Introduction

Pain is a very common concern for patients and has a significant effect upon their quality of life. Specifically, pain, as defined by the International Association for Pain Research, is an unpleasant feeling and emotional experience associated with potential or existing tissue damage. (Treede, 2018). Pain can be divided into acute and chronic pain depending upon its duration. Chronic pain, which is defined as pain lasting for longer than 3 months after the onset of the initial injury or disease, affects the quality of daily living (Mills et al., 2019). Chronic pain is challenging to treat due to the limited efficacy and adverse side effects of therapies. Commonly used analgesics are classified as opioids (cocaine, morphine) and nonsteroidal anti-inflammatory drugs (NSAIDs), which have a good clinical therapeutic effect. However, opioids carry a significant risk of severe side effects including nausea, itching, sedation, respiratory depression, addiction, tolerance and dependence, and even the unacceptably high death rate for repeated dosing of opioids (Lutfy, 2020; Uniyal et al., 2020; Seth et al., 2021). According to the survey, in 2016, around 2.1 million Americans were addicted to opioids, and more than 42,000 patients died due to opioid overdose (Seth et al., 2018). Therefore, clinical guidelines discourage the use of opioids to treat chronic pain (Edelman and Hemmert 2019) and the exploration and discovery of new types of analgesics with little adverse reactions, highly safe and with good selectivity are urgently needed.

Ginseng (Panax ginseng C.A. Meyer) is an herbal plant within the Araliaceae family and belongs to the Panax Genus (Guo et al., 2021). The roots of ginseng contain ginsenoside, a class of steroid glycoside that is responsible for its pharmacological activity. Ginseng has many bio properties such as antioxidant (Chen and Huang, 2019), anti-inflammatory (Im, 2020), analgesia antipruritic (Lee et al., 2018; Luo et al., 2018), and anticancer activities (Zhang J. et al., 2019). Previous phytochemical and pharmacological investigations have also demonstrated antinociceptive effects of ginseng extracts in various pain models including those in relation to the abdomen (Wang et al., 2018), neuropathic pain (Lee et al., 2021b), chronic joint pain (Chen et al., 2014), and incisional pain (Kim et al., 2018). The mechanisms of action suggested to explain these effects include antagonism of adrenergic (Kim et al., 2018), gamma-aminobutyric acid (Yin et al., 2011), and opioid receptors (Suh et al., 2000); and regulation of ion channel activity (Lee, Kim, and Shim 2018), mediation of pro-inflammatory cytokine expression (Luo et al., 2018), and immune cell responses (Chen et al., 2014).

In this review, we provide an overview of ginseng and its active constituents and its major therapeutic applications for the treatment of chronic pain (neuropathic pain, inflammatory pain, osteoarthritis pain, abdominal pain, etc.). We discuss neurobiological and pharmacological mechanisms of action of ginseng and ginsenoside’s analgesic effects on chronic pain.

## Chemistry of Ginseng and Effect of Ginsenosides

Ginseng is mainly composed of ginsenosides, polysaccharides, proteins, amino acids, volatile oils, and lignin (Huang et al., 2021). Among these components, ginsenosides have been implicated as the bioactive constituents, which can be divided into protopanaxadiol (with no hydroxyl group at position six), protopanaxatriol (with a hydroxyl group present at position six) (Table 1), oleanolic acid, and ocotillol types, all of which have different effects. Protopanaxadiol mainly includes the ginsenosides Rb1, Rb2, Rb3, Rc, Rd, Rg3, and Rh2 and compound K, whereas protopanaxatriol is mainly composed of the ginsenosides Re, Rg1, Rg2, and Rh1. These types of ginsenosides have different biological activity and different clinical effects, such as regulation of the central nervous system (Chen et al., 2019; Park et al., 2012b; Wu et al., 2018), promoting learning and memory ability (Chen et al., 2018; Jiang et al., 2021), improving heart function (Li X. et al., 2020; Lu M. et al., 2021), hypoglycemic effects (Tabandeh et al., 2015; Meng et al., 2017), improving resistance to stress (Chen et al., 2016; Li c. w. et al., 2020), and modulating the immune response (Park et al., 2015). Based on various clinical studies, the pharmacokinetics of the different types of ginsenosides remain unclear because of their heterogeneous chemical structures (Qi et al., 2011). Here, we highlight their roles and mechanisms of action in the regulation of chronic pain.

**TABLE 1:** Classification of ginsenosides and their effects.

Type	Chemical formula	Ginsenoside	Effects	References
Protopanaxadiol type	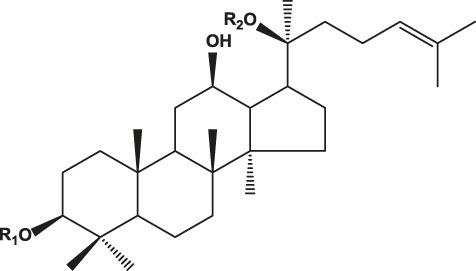	Ra1, Ra2, Ra3, Rb1, Rb2, Rb3, Rc, Rd, Rg3, Rh2, F2, R1	Diverse anticancer activities; Regulating central nervous system; Improving learning and memory; Analgesic effect	Chen et al. (2018), Jiang et al. (2021), Kim et al. (2018), Park et al. (2012b), Sun et al. (2019), Wang et al. (2021), Wu et al. (2018)
Protopanaxatriol type	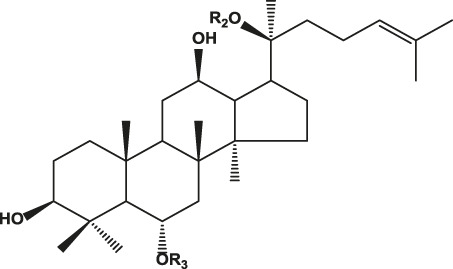	Re, Rf, Rg1, Rg2, Rh1, R1, R2, F1	Improving heart function; Inhibiting the growth of tumor cells; Anti-inflammatory Relieve neuropathic pain	Huang et al. (2019), Kim et al. (2012), Li C. W. et al. (2020), Li et al. (2019), Lu et al. (2021), Zhang Q.-L. et al. (2019)
Oleanolic acid type	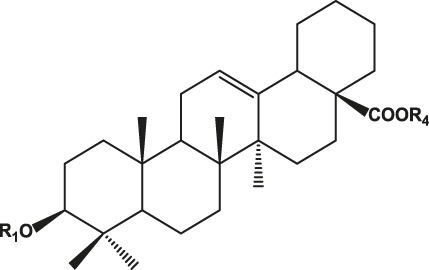	R0, Rh3, Ri	Anti-inflammatory; Anti-thrombotic effect	He et al. (2013), Hu et al. (2008), Lee et al. (2015), Lin et al. (2016)
Ocotillol type	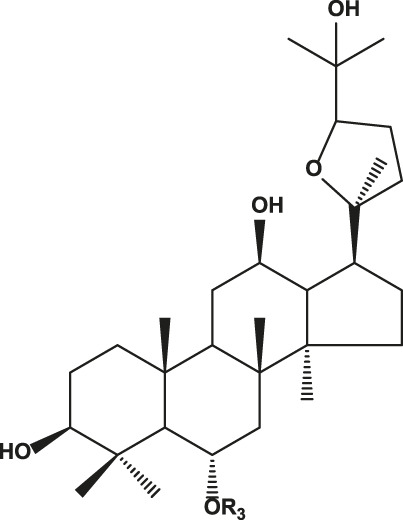	R2, Rg5	Inhibiting the growth of tumor cells; Proliferation and apoptosis of cancer cells	Dong et al. (2019), Jeong et al. (2015)

R1: Glc-Glc- or Glc- or H.

R2: Rha-Glc-O- or Glc-Glc-O- or Glc-O- or H or OH.

R3: Glc-Glc- or A-Glc- or Glc- or H.

R4: Glc-.

## Ginseng in Chronic Pain

Ginseng has been widely used to modulate various types of chronic pain such as neuropathic pain, inflammatory, and other types of chronic pain. At least 10 of ginseng saponins could inhibit NF-κB transcription factor (Kim et al., 2009) as well as reduce inflammatory NO synthase expression (Huynh et al., 2020), while some ginsenosides have direct effect against neuropathic pain that blocks calcium channels in nociceptive neurons (Mogil et al., 1998). In this section, we will introduce the effects of ginseng and ginsenosides on variety of chronic pain and its mechanism.

### Ginseng in Neuropathic Pain

Neuropathic pain is a type of chronic pain that arises as a direct consequence of a lesion or disease affecting the somatosensory system. Approximately 7–10% of the population suffers from pain with neuropathic characteristics (Wright and Rizzolo, 2017). The pathogenesis of neuropathic pain is complicated, spinal cord injury (Lee et al., 2021b), immune diseases (Lu H. J. et al., 2021), diabetes (Rosenberger et al., 2020), and cancers (Fallon, 2013) can all cause neuropathic pain. The effect of traditional opioid analgesics and non-steroidal anti-inflammatory drugs on neuropathic pain are less than ideal. Furthermore, long-term use of opioids are potentially very addictive and can cause other side effects. The bioactive components of ginseng have been reported to possess certain therapeutic effects on neuropathic pain. Recently, Lee et al. (2019) evaluated the effects of ethanol extracted white ginseng (GS-KG9) on streptozotcin (STZ) induced diabetic neuropathic pain in rats. They found that oral administration of GS-KG9 lowered blood glucose levels and inhibited microglial activation in position L4–L5 of the spinal cord in STZ-induced hyperglycemic rats. This analgesic effect of GS-KG9 was found to be mediated by the inhibition of microglial activation through reducing blood glucose levels. GS-KG9 also inhibits hyperexcitability of the spinal cord dorsal horn neurons thus attenuating allodynia and hyperalgesia. Kim et al. (2020) reported that intrathecally administered Korean red ginseng produced a dose-dependent antiallodynic effect in a chemotherapy-induced neuropathic pain model. Furthermore, 5-Hydroxytryptamine (5-HT) receptor antagonists reversed this effect. Their results suggested that red ginseng is effective against chemotherapy-induced neuropathic pain at the spinal level through mediating 5-HT receptor expression. In addition, Suzuki et al. (2017) found that ginseng extracts showed a protective effect against neurite damage induced by oxaliplatin and relieved oxaliplatin induced neuropathic pain in mice. Furthermore, treatment with ginseng extract significantly ameliorated both cold allodynia and mechanical hyperalgesia induced by oxaliplatin, whereas oxaliplatin treatment suppressed neurite outgrowth from primary dorsal root ganglia, and ginseng extract inhibited this suppression. These results suggest that ginseng extract could be an effective agent for the treatment of oxaliplatin-induced neuropathic pain. Lee et al. (2021b) reported that the ginsenoside Rb1 and compound K released peripheral and central neuropathic pain through estrogen receptors. Activation of microglia/astrocytes and the expression of inflammatory mediators such as IL-1β, iNOS, and Cox-2 were also significantly inhibited in the L4–L5 spinal cord of central neuropathic rats by oral administration of Rb1 and compound K. However, the antinociceptive effects of Rb1 and compound K were reversed by treatment with estrogen receptor (ER) antagonists. These results indicated that Rb1 and compound K have potential antinociceptive effects against both peripheral and central neuropathic pain that may be mediated through the estrogen receptor. Li et al. (2019) reported that ginsenoside Rf can effectively decrease pain hypersensitivity, depression-like behavior and inflammatory reactions in chronic constriction injury induced neuropathic pain model. Chronic treatment of ginsenoside Rf partially inhibits increase of proinflammatory cytokines in the injured DRG and spinal cord. However, it promotes the IL-10 expression, an anti-inflammatory factor. Those results suggest ginsenoside Rf alleviates neuropathic pain and its associated depression by restoring the balance between proinflammatory and anti-inflammatory cytokines.

### Ginseng in Inflammatory Pain

In many types of chronic pain, inflammatory pain represents a major component. Inflammatory pain is defined as increased sensitivity caused by an inflammatory response associated with tissue damage (Muley et al., 2016). When inflammatory pain occurs, an innocuous stimulus, which usually would not cause pain can be perceived as painful. This kind of primary hyperalgesia is a direct consequence of peripheral nerve sensitization mediated by inflammatory mediators and other factors such as serotonin, protons (change in pH), bradykinin, and cytokines. Recent findings suggest that ginseng has a positive effect on anti-inflammatory pain, and Jang et al. (2016) reported that the ginsenoside Rb1 attenuates acute inflammatory pain by inhibiting neuronal extracellular signal-regulated kinase (ERK) phosphorylation via regulation of the Nrf2 and NF-κB pathways. The authors used formalin to induce inflammatory pain in rats and found that intrathecal injection of Rb1 could effectively relieve the formalin-induced acute inflammatory pain response. Furthermore, they also confirmed this analgesic effect was caused by the inhibition of ERK phosphorylation and microglial (astrocyte) activation through regulation of the nuclear factor erythroid 2-related factor 2 nuclear factor kappa B pathway in the spinal cord. Taherianfard and Aalami (2020) also reported that ginseng extract decreased pain sensitivity through regulation of dopamine D2 receptor activity in this formalin model. Furthermore, Nah et al. (2000) investigated the analgesic effect of ginsenosides on a capsaicin-induced inflammatory pain model and found that either intraperitoneally or intrathecally administered ginsenosides suppressed capsaicin-induced nociceptive behavior in a dose dependent manner and that administration of ginsenosides did not significantly affect the motor ability of the animal. Their results suggest that ginsenosides produced antinociceptive effects through their action at spinal or supraspinal sites, not at peripheral nociceptors or opioid receptors.

### Ginseng in Other Chronic Pain Conditions

Ginseng is not only effective in the treatment of neuropathic and inflammatory pain, but also in other types of chronic pain. Osteoarthritis is an age-related degenerative disease, often resulting in chronic joint inflammation and pain. The most common medications used to treat Osteoarthritis are NSAIDs, which increase the risk for gastrointestinal distress (Rannou et al., 2016). Several researchers have also reported ginseng and ginsenosides as having analgesic effects on osteoarthritis induced chronic joint pain. Choi et al. (2013) reported that compound K decreased the production of matrix metalloproteinases (MMPs) from fibroblast like synoviocytes by inhibiting Janus kinase (JNK) and ERK signaling pathways and Kim et al. (2007) evaluated the antiarthritic effects of ginsenoside Rb1 in collagen-induced arthritic mice. Their results suggested that oral administration of ginsenoside Rb1 significantly reduced clinical arthritis scores and decreased immune cell infiltration and cartilage destruction. Treatment with ginsenoside Rb1 suppressed TNF-α expression, which was upregulated during inflammatory responses in collagen-induced arthritic mice.

Incisional hernias represent a very common clinical symptom after surgical incision or intraoperative injury and Kim et al. (2018) reported that ginsenoside Rf had significant antinociceptive and anti-inflammatory effects on an incisional pain model in rats. Ginsenoside Rf increased the mechanical withdrawal threshold significantly, with a curvilinear dose–response curve peaking at 1.5 mg/kg and the levels of IL-1β, IL-6, and TNF-α significantly decreased after ginsenoside Rf treatment. Interestingly, the antinociceptive effect of ginsenoside Rf was reduced by yohimbine, but potentiated by prazosin and ketanserin, suggesting that adrenergic receptors might be involved in pain regulation by ginsenoside Rf. Irritable bowel syndrome (IBS) is a chronic gastrointestinal disorder associated with abdominal pain and irregular bowel habits such as constipation and diarrhea. Currently, IBS is recognized to be a multifactorial disorder with a heterogeneous and complex pathophysiology. Thus, It is difficult to develop safe and effective therapy (Chey et al., 2015). Ginseng as a traditional health care medicine is also used to treat IBS in Asia. Kim et al. (2005) evaluated the effect of ginsenosides on a rat model of IBS and found that they inhibited 5-HT3A receptor channel activity. Furthermore, because the 5-HT3A receptor is closely related with IBS in enteric nervous system, oral administration of ginsenosides significantly and dose-dependently inhibited acetic acid-induced visceral hypersensitivity. A clinical study reported (Rocha et al., 2018) in Brazil also showed that the dry extract of ginseng could reduce abdominal pain in IBS patients. Clearly, from the above-mentioned pain causes ginseng and ginsenoside have a good therapeutic effect. In addition, many studies have reported that ginseng and ginsenosides also have effects on bone cancer pain, cancer pain, and migraine. As further research and clinical applications are increasing, it is clear that ginseng shows great potential as a future therapy for the treatment of chronic pain, or as an adjunct treatment for multiple pain types.

## Mechanism of Action, Target and Signaling Pathways

**FIGURE 1:**
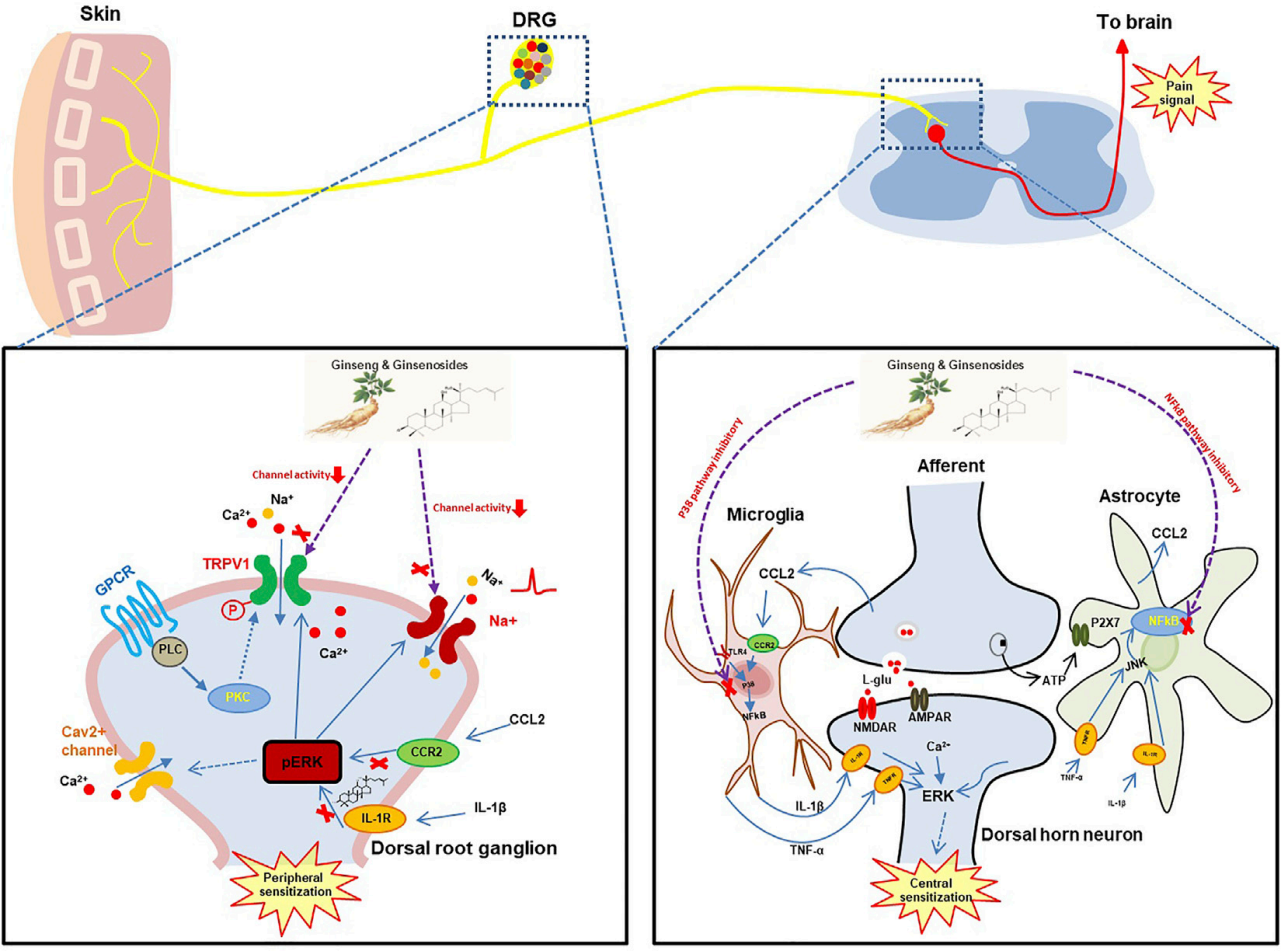
The hypothetical analgesic action mechanisms of ginseng and ginsenosides. DRG, dorsal root ganglion; TRPV1, transient receptor potential cation channel subfamily V member 1; GPCR, G protein-coupled receptor; PLC, phospholipase C; PKC, protein kinase C; CCR2, C-C motif chemokine receptor 2; CCL2, C-C motif chemokine ligand 2; TLR4, Toll-like receptor 4; JUK, Janus kinase; NMDAR, N-methyl-D-aspartate receptor;AMPAR, α-amino-3-hydroxy-5-methyl-4-isoxazolepropionic acid receptor.

Chronic pain is maintained by both central sensitization and peripheral sensitization, where neuronal responsiveness increases, thus presenting the phenomenon of synaptic plasticity (Figure 1). With the advent of noxious stimuli, neuroinflammation is triggered causing the activation of glial cells in dorsal root ganglia, spinal cord, and brain which drives the production of pro-inflammatory cytokines and chemokines in the PNS (peripheral sensitization) and CNS (central sensitization) (Ji et al., 2018; Matsuda et al., 2019). Studies have found that ginseng not only plays an important role in the anti-inflammatory process but reduces hypersensitivity in neurons. It also helps to enhance inhibitory circuitry in pain transduction and bridge the gap between microglia and neurons by regulating inflammatory molecules. Hence now we will focus on the antinociceptive mechanism of ginseng on specific targets.

### Microglia

Microglia are a group of resident macrophages originating from the yolk sac and belong to mononuclear phagocyte system, where they play a role in immune surveillance in the CNS (Suzumura 2013; Nayak et al., 2014). Microglia remain inactive under normal physiological states, but then become activated during injury or during a pathogen invasion. Evidence suggests that microglia represent key players in the development and maintenance of chronic pain (Ji et al., 2013). Depending upon the type of stimulus, normally lipopolysaccharide (LPS) or IL-4, microglia can be classified into classical activation state M1 as a pro-inflammatory state which also has neurotoxic effects, as well as an alternative activation state M2 involved in neuroprotective process (Hernandez Baltazar et al., 2020; Orihuela et al., 2016; Tu et al., 2021).

Though the activation of microglia can be beneficial during the recognition of pathogen or abnormal protein aggregation, inducing cellular phagocytosis, the persistent activation of microglia generates hyper-neuroinflammation which then facilitates hyperexcitability of pain signaling (Tiwari et al., 2014).

The multiple constituents of ginseng including the ginsenosides Rb1, Rb2, Rg1, Rg3, Re, Rh1, Rh2, and its metabolite compound K inhibit the activation of microglia, thereby ameliorating inflammatory cellular pathways such as NF-κB signaling (Jung et al., 2010; Park et al., 2012; Park et al., 2012a; Shi et al., 2019; Wu et al., 2007; Xu et al., 2020; Yao et al., 2019). For example Rg1 can attenuate the expression of iNOS, COX-2, TNF-α, IL-1β, and NF-κB in LPS-induced BV-2 microglial cells via the activation of the PLC-γ1 signaling pathway, and this involves the phosphorylation of IκB-α, CREB, ERK1/2, JNK, and p38 MAPK (Zong et al., 2012). Furthermore, the ginsenoside Rg1 can mediate the expression of neuroinflammatory biomarkers and inhibit chemotherapy-induced microglial polarization from M2 to M1 phenotypes (Shi et al., 2019). A further mechanism involving the ER has been reported in a recent study. Administration of Rb1 has been found to alleviate allodynia and hyperalgesia in central neuropathic pain-induced rats by inhibition of microglia in the L4–L5 spinal cord. These antinociceptive effects of Rb1 can be reversed by ER antagonist, indicating that the antinociceptive mechanism of action of ginseng is clearly complex in microglia and may involve several intracellular pathways and therefore further investigation is needed its full elucidation (Lee et al., 2021a).

### Neurons

The crosstalk between neurons and microglia involving cytokines and chemokines represents one aspect of pin sensitization (Figure 1). However, multiple ion channels such as voltage-gated Na^+^ channels and voltage-gated Ca^2+^ channels localized in primary sensory neurons remain a key component in the perception of pain associated with the pathogenesis of chronic neuropathic and inflammatory pain conditions (Lee et al., 2021a).

A polyacetylenic compound isolated from ginseng extract has been found to inhibit Na^+^ currents and can accelerate their inactivation, thus producing a hyperpolarizing shift of the steady-state inactivation curve in acutely dissociated rat dorsal root ganglion (DRG) neurons (Choi et al., 2008). Similarly, application of ginseng total saponins, in particular ginsenoside Rg3, suppresses high-voltage-activated Ca^2+^ channel currents via pertussis toxin-sensitive G-protein pathways similar to that seen for μ-opioid receptors (Rhim et al., 2002). In addition, red ginseng extract has an inhibitory effect on histamine-induced itch sensation by inhibition of the H1R/TRPV1 ion channel present in DRG neurons (Jang et al., 2015). Noteworthy, TRPV1 is a non-selective cation channel highly expressed in DRG neurons and the spinal cord, believed to serve as a viable target in the regulation of chemotherapy-induced neuropathic pain (Akhilesh et al., 2022). Rg1 blocks intracellular calcium by both capsaicin and proton activation in a TRPV1-dependent manner indicating its potential as an antagonist of TRPV1 (Huang et al., 2012). However, it seems that the antinociceptive effect of ginseng is more potent in the central than peripheral nervous system. Intrathecal administration of ginsenosides was found to intracerebroventricularly suppress capsaicin-induced pain-related behavior in mice rather than when administered subcutaneously. Ginsenosides can also inhibit excitatory neurotransmitter release from primary sensory nerve terminals following capsaicin injection independently of opioid receptors (Nah et al., 2000). The same group also finds that ginsenoside Rc enhanced capsaicin-induced inward currents in Xenopus oocytes expressing the vanilloid receptor in a concentration-dependent and reversible manner. This suggests a different channel is utilized by ginsenosides to regulate capsaicin-induced pain in DRG neurons (Jung et al., 2001).

A recent study has reported that ginseng influences the release of dopamine through the cholinergic system, and directly affects dopamine D2 receptors to exert its analgesic effect. Application of ginseng extract alone or with D2 receptor agonists synergistically has an obvious analgesic effect with no additive effect (Taherianfard and Aalami 2020). Taken together these findings indicate that the antinociceptive mechanism of action of ginseng between neurons could be extensive.

The N-methyl-D-aspartate (NMDA) receptors are excitatory glutamate receptors and widely expressed in the pain transmission pathway whose hyperactivity fires pain signal projecting to the supraspinal structures of the CNS, making it a progress to central sensitization (Uniyal et al., 2021). The weak efficacy of traditional NMDA antagonists such as ketamine and memantine applicated in pain management suggests the urgent needs for development of new NMDA receptor antagonists for analgesia (Kreutzwiser and Tawfic, 2019; Uniyal et al., 2021). Intraperitoneal injection of ginseng total saponins in hyperalgesia model significantly increases mechanical withdrawal threshold, and this antinociception of ginseng total saponins is reserved by additional injection of NMDA (Kim et al., 2014). Ginsenoside Rg3 and Rk1 effectively antagonize NMDA receptors in rat neurons through respective mechanisms, indicating the potency of ginsenosides as NMDA antagonists in pain management (Ryoo et al., 2020).

### Other Potential Targets

When compared to microglia, little work has been reported concerning the effects of ginsenosides in other glial cells including astrocytes and oligodendrocytes, both important in central sensitization (Donnelly et al., 2020; Kwon and Koh, 2020). Based on current findings, ginseng functions to protect astrocytes from hypoxia or oxidant stressors (Naval et al., 2007; Choi et al., 2019), facilitating clearance of extracellular glutamate (Zhang et al., 2013), inducing astroglial autophagy (Rahman et al., 2020) and inhibiting their activation during ischemia and mild stress (Fan et al., 2018; Liu et al., 2020). Gintonin facilitates late differentiation of oligodendrocytes in primary oligodendrocyte precursor cells, suggesting a potential new treatment for demyelinating diseases (Mijan et al., 2019). Schwann cells are important glial cells in the PNS, and provide neurotropic support for peripheral nerves after injury and can interact with nociceptive neurons to regulate different pain conditions (Wei et al., 2019). Ginseng and Rg1 enhance proliferation of RSC96 Schwann cells in a dose-dependent manner by increasing the phosphorylation of ERK, NH2-terminal JNK, and p38, which are sub-families of mitogen-activated protein kinases (MAPKs). In addition, ginseng and Rg1 also stimulate the FGF-2-uPA-MMP-9 migration pathway to enhance the migration of RSC96 Schwann cells (Lu et al., 2009). Similarly, Rb1 and Rg1 promotes secretion of nerve growth factor and brain-derived neurotropic factor in cultured Schwann cells mainly through the protein kinase A (PKA) pathway (Liang et al., 2010). Wang et al. reported that Re also promotes Schwann cell proliferation, differentiation, and migration *in vitro* via activating ERK- and JNK-signaling (Wang et al., 2015). As mentioned above, like microglia, macrophages have pro-inflammatory M1-like and anti-inflammatory M2-like phenotypes, and their cytokine pattern of release can influence pain transduction and neuronal conduction via modulation of ion channels (Ji et al., 2016). Ginseng promotes the polarization of M2 to M1 phenotype and increases the production of immunomodulators such as TNF-α, IL-1β, IL-6, NO, iNOS, and COX-2, which causes enhanced macrophagic phagocytosis of bacteria whose components such as LPS produce pain hypersensitivity in nociceptors (Cao et al., 2019; Xin et al., 2019; Um et al., 2020). From these findings further research remains necessary into the direct/indirect analgesic role of ginseng and its ginsenosides in pain modulation and in the intricate pain sensitivity loop between glial cells and immune cells both in the PNS and CNS.

## Conclusion

Chronic pain is typically characterized by peripheral mechanical allodynia and heat hyperalgesia. Opioids and NSAIDs are well-established pharmacological therapies for the treatment of chronic pain. However, these drugs have not always been effective against chronic pain and have some side effects because of their binding to off-site targets. The potential analgesic effects of ginsenosides on chronic pain are very clear and effective. Recent studies have focused upon the effects of natural anti-inflammatory drugs such as ginsenosides on chronic pain treatment, largely due to their relative safety, reliability, and affordability. The molecular and cellular mechanisms of action of ginseng and its bioactive constituents the ginsenosides include the modulation of neurotransmitter function in both peripheral and central systems, inhibition of inflammatory cytokine expression, modulation of ion channel activity in DRG and spinal cord neurons, regulation of the TLR4/NF-κB signal transduction axis, and anti-inflammatory effects. This review has provided a theoretical basis and clinical application for the treatment of chronic pain based on Panax ginseng. Hence, ginsenosides have great potential for future pain treatment or as an adjuvant for pain therapy in multiple pain types.

## Abbreviations

NSAIDs, nonsteroidal anti-inflammatory drugs; 5-HT receptor, 5-hydroxytryptamine receptor; ER, estrogen receptor; JNK, janus kinase; ERK, extracellular signal-regulated kinase; MMPs, matrix metalloproteinases; IBS, irritable bowel syndrome; PNS, peripheral sensitization; CNS, central sensitization; LPS, lipopolysaccharide; IFN-γ, interferon-gamma; GM-CSF, granulocyte-macrophage colony-stimulating factor; MAPKs, mitogen-activated protein kinases; STZ, streptozotocin; PKA, protein kinase A.
